# Intraoperative Patella Dimension Measurement in Asian Female Patients and Its Relevance in Patellar Resurfacing in TKA

**DOI:** 10.1155/2020/4539792

**Published:** 2020-04-25

**Authors:** Shaifuzain Ab Rahman, Amran Ahmed Shokri, Muhammad Rajaie Ahmad, Ahmad Filza Ismail, Nur Syahida Termizi

**Affiliations:** ^1^Department of Orthopedic, Hospital Universiti Sains Malaysia, 16150 Kubang Kerian, Kelantan, Malaysia; ^2^Department of Community Medicine, Hospital Universiti Sains Malaysia, 16150 Kubang Kerian, Kelantan, Malaysia

## Abstract

**Background:**

Implants used for total knee arthroplasty (TKA) in Asian patients are mostly produced based on anthropometry of the Western population, thus causing problem with patella sizing, especially in Asian females where the patellae are regarded to be smaller. This study is to define intraoperative patella dimensions in our female populations and compare them with current prosthetic systems available at our institution.

**Methods:**

This is a cross-sectional study involving 156 TKA female patients with normal patellae. The patella height, width, thickness, medial and lateral articular facets' width and thickness, and the dome position were measured. The smallest implant size from 3 manufacturers was compared to the data obtained. Analysis using descriptive statistics was used to get the mean and median of anatomical patella dimensions, whereas the independent *T* test and one-way ANOVA test were used to compare the Malaysian female's patella dimensions with various implant sizes.

**Results:**

The articular surface of the patella was found to have an oval shape with a width-height ratio of 1.31. The mean (SD) patella thickness, width, and height were 20.7 (1.85) mm, 40.7 (3.79) mm, and 31.3 (2.81) mm, respectively. Only 17.9% fit for smallest implant size from all 3 manufacturers. The oval-shape implant was suitable in 53.8% patients based on their width-height ratio. The dome position is 2.2 mm medial to centre.

**Conclusion:**

These female patients have thinner and smaller patella, which are generally unable to accommodate patellar components based on the Caucasian database. Therefore, orthopaedic implant manufacturers should consider optimizing the thicknesses as well as widths of their patellar prostheses.

## 1. Introduction

The patella is the largest sesamoid bone, which shields over the anterior surface of femoral condyles [1]. Its posterior surface can be divided into superior articulating surface and inferior nonarticulating surface. A vertical ridge further subdivides the superior part into medial and lateral facets. Wiberg has classified patella into 3 types, which is based on the width dimensions of the medial (MAF) and lateral articular facets (LAF) [2].

The patella is a part of the patella-femoral joint, which is essential to provide knee stability through its role in the extensor mechanism, whereby it improves mechanical property of extensor muscles. This is conducted via transmitting the extensor force across the knee at a greater distance from the centre of rotation. The magnification of moment arm reduces quadriceps force to extend the knee by 15% to 30% [3, 4].

Patella replacement in total knee arthroplasty (TKA) is not a universal procedure for all arthroplasty surgeons; thus, ample knowledge on its science is required. This includes knowledge of the patella anatomy and understanding the pathological changes that affect it [5]. Anthropometric measurements of the patella and patella ligament are crucial in the diagnoses and surgical corrections of knee-related injuries [4, 6].

A success in functionality of TKA with patella replacement highly depends on appropriate size and thickness of the chosen patella implant [6]. Poor design characteristics of the implants and technical errors were related to early patellar failures in a tricompartment TKA [7].

Various patella components are currently available in the market, which allow the prostheses to be onset or inset by using symmetrical or asymmetrical domes [7]. Most systems highlight on replacing the articular surface, correct positioning of the dome, and restoring the patella thickness. However, they do not focus on maximizing the anatomic bony coverage of the patella [7].

Implant components used for TKA in Asian patients are mostly produced based on anthropometry of the Western population. There would be differences in terms of conformity of implants to the patient's anatomy and clinical results after TKA due to anatomic features and life styles dissimilarities between Western and Asian populations. For that reason, surgeons in Asia are particularly concerned in related surgical techniques and implant designs that are being used in TKA for better quality clinical results as well as patient satisfaction [8, 9].

This study was conducted to define intraoperative anatomy of patella dimensions in female patients undergoing TKA and compare them with the commonly used prosthetic component systems. The goal is to get practical anatomic patella data in our female population. It is also to help in the development and improvement of patella component design and implantation for TKA in this population.

## 2. Materials and Methods

This was a cross-sectional study carried out at our centre for a period of 12 months from July 2016 to Jan 2018. Ethical approval from the institutional review board (IRB) was obtained before the study was conducted. All female patients who underwent TKA at our institution during this period of study were recruited with exclusion. We excluded patients with severe patella deformity from previous trauma, severe osteoarthritis of patellofemoral joint, or congenital deformity that normal anatomy could not be determined.

Initial detailed and thorough explanation regarding the TKA procedure was carried out by an arthroplasty surgeon during preoperative consultation at the orthopaedic clinic of our institution. Verbal and written consents from participants were obtained prior to the TKA.

Intraoperative anatomic measurement of patella was carried out by one of the two arthroplasty surgeons by using a surgical vernier caliper which is sensitive up to 0.1 mm.

**Figure 1:**
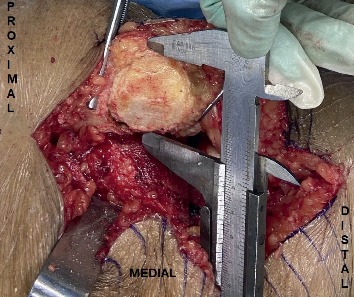
Measurement of left patella's width using a vernier caliper.

**Figure 2:**
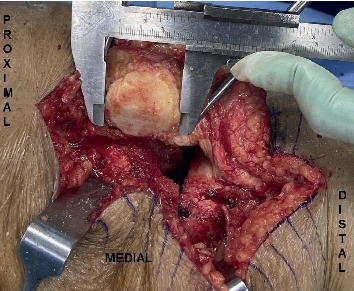
Measurement of left patella's height using a vernier caliper.

The patella dimensions were measured after the marginal osteophytes were properly removed. The dimensions were inclusive of the patella height, which was from the edge of the superior articular surface to the margin of the inferior articular surface; the patella width, which was from the border of the medial articular surface to the border of lateral articular surface; and the patella thickness, which was the maximum thickness measured at the centre of the dome [7]. Finally, the widths of medial articular facet (MAF) and lateral articular facet (LAF) as well as their thickness were also measured (Figure 1 and 2). The average time taken for the measurement was less than 5 minutes, which was added to the normal time taken for TKA procedure. All measurements were recorded in the data collection sheet (Study Proforma).

The anthropometric data obtained intraoperatively were compared with the smallest patella component size from 3 commonly used manufacturers at our centre.

Data were analyzed using software IBM SPSS version 22 including descriptive statistics and compared the means using the independent *T* test and one-way ANOVA. Confidence interval was set at 95%, and *p* value <0.05 was considered statistically significant.

## 3. Results

There were 128 female patients undergoing 186 TKAs during the period of this study. However, following our inclusion and exclusion criteria, we ended up collecting data from 156 patellae of 102 patients who underwent TKA. The patients were between 45 to 79 years old with a mean age of 62.5 years old (SD 7.41). Out of 156 patients, 144 (92.3%) are Malays, 9 (5.8%) are Chinese, and 3 patients (1.9%) are from Indian ancestry.

The patella thickness ranges from 18 to 26 mm with mean 20.74 (1.85) mm. Patella height ranges from 22 to 38 mm with mean 31.33 (2.81) mm. Patella width ranges from 31 to 51 mm with mean 40.76 (3.79) mm. MAF width ranges from 13 to 27 mm with mean 19.38 (2.70) mm. LAF width ranges from 15 to 28 mm with mean 21.30 (2.27) mm. MAF thickness ranges from 11 to 20 mm with mean 15.90 (2.24) mm. LAF thickness ranges from 12 to 22 mm with mean 16.93 (2.16) mm. Width-height ratio ranges from 0.97 to 1.68 with mean 1.31 (0.13). These results are summarized in Table 1.

Out of 156 patients, 82 (52.6%) were measured from right knees, while 54 (47.4%) were from left knees. Based on Wiberg Classification [3], 94 (60.3%) patellae were type 1 patella, 53 (34.0%) were type 2, and 9 (5.7%) were type 3 patella. The mean dome position is 2.2 mm (3.4 mm) medial to the centre of the patella. This is consistent with the overall finding of Wiberg type 1 in this population.

The mean patella dimensions including patella height, patella width, patella thickness, MAF width, and LAF width between right side and left side was not statistically significant different (*p* > 0.05) (Table 2). The mean patella thickness and patella width between Wiberg types were also not statistically different (*p* value >0.05) (Table 3).

Based on the measurement of patella dimensions, further analysis comparing patients' patella with the smallest patella components from the 3 commonly used manufacturers were carried out. The smallest patella size (diameter) for implants A and C is 30 mm. The smallest size for implant B is 32 mm. Out of 156 patellae, 66 (42.4%) patellae fit for the smallest implant size from manufacturers A and C, while only 28 patellae fit for the smallest implant size from manufacturer B. Thus, there were 90 (57.6%) patellae that did not fit all smallest implants (A and C) used at our centre.

Based on their width-height ratio, there were 84 patellae (53.8%), which were suitable for oval-shape implants, while another 72 (46.2%) were suitable for round-shape implants. The mean width-height ratio between group of patients suitable for oval-shape implants and round-shape implants was statistically significant different with *p* value <0.001.

The mean patella height between the group of patients suitable for oval-shape implants and round-shape implants was statistically significantly different (*p* value <0.001). The mean patella height of patients suitable for round-shape implants was higher compared to the mean patella height of patients suitable for oval-shape implants, with 2.50 mean difference between these two groups (Table 4).

## 4. Discussion

A success in functionality of knee arthroplasty is highly dependent on appropriate size and thickness of the chosen patella implant [6]. This includes avoiding overstuffing or oversizing of the patella. This study aimed to document the anthropometric measurement of patella dimensions among our female patients receiving TKA and compare them with the available current patella implants size.

We only include female patients in this study since majority (>89%) of TKA patients at our centre are females. In addition, following previous studies, female is expected to have smaller patella dimensions [6, 7, 10–12]. Therefore, it is appropriate to assess if this female population will have issue fitting the smallest available patella component from the 3 commonly used TKA systems.

In the present study, the mean patella thickness, patella width, and patella height were 20.7 mm, 40.7 mm, and 31.3 mm, respectively. In an American study [7], the mean patella thickness was 21.8 mm, the mean patella width was 42.7 mm, and the mean patella height was 35.0 mm in their female samples. Another study among the South African females with European ancestry showed the mean patella thickness, patella width, and patella height were 22.8 mm, 42.7 mm, and 41.0 mm, respectively [11]. Based on the above data, it is concluded that our female population have thinner and smaller patellae than the Westerners.

In this study, Wiberg type I patellae, in which the MAF and LAF widths are concave and equal, were the most prevalent with 60.3% of the patients as compared to 34% of Wiberg type II patellae. This is different from the study by Wiberg himself in 1941 that reported type II patellae were the most prevalent [2]. This finding was supported by Fucentese et al. which also showed Wiberg type II as the majority in his series [13].

The average dome of the articular surface of the patella in this study is located at 2.2 mm medial of centre, which is consistent with Wiberg type I patellae. Aglietti et al. [14] showed that, in their series, the mean dome position was 4.0 mm medially. While in the Hofmann et al. [15] series, the mean dome was 5.4 mm medially. These patellae are consistent with Wiberg type II or III. Baldwin and House series has the closest mean dome position (at 3.7 mm) to this study's findings [7]. However, the series still showed predominantly Wiberg II patella in their study. Based on the above findings, previous studies had agreed on the benefits of medialization of the patella during the patellar resurfacing [15, 16]. However, in our series, medialization of the patella is not usually necessary since most of our patellae are Wiberg I.

In patellofemoral arthritis, the chondral wear is the most prominent in the lateral patellar facet, which points out that the lateral patellar facet receives higher load than the central or medial aspect of the patella [13]. Since majority of our patients had almost equal MAF and LAF widths, the higher load exerted on lateral facet may lead to a higher risk of developing patellofemoral arthritis in our population. Another supportive evidence is that the highest compressive load is found primarily beneath the lateral facet, which are related to the higher subchondral bone densities [17]. It has been described that the patellar trabecular bone architecture over the lateral facet is nonhomogenous stacking up of sheets resulting from improved remodelling process because of the constant pressure in situ [18].

This current study shows moderate to good correlation between patella width and patella thickness, which is consistent with the results of Iranpour et al. [10] and Olateju et al. [11]. This finding explains that, in general, smaller patellae will not only have problem fitting the implant but also face problem to maintain minimum bone thickness upon resection. Patella width has been proposed to be a dependable factor for predicting the normal size of patella thickness, which helps the surgeons to decide on the thickness of the patella prosthesis during TKA [10].

This study also shows that 90 patients (57.6%) do not fit the smallest available patella component (size 30 mm) used at out institution. Although we did not have patella component smaller than size 30 mm during the period of this study, we further analyzed the number of patients that would fit smaller component sizes. For a size 28 mm, 101 (64.7%) of our patellae fit the patella component. Furthermore, the theoretical analysis shows that a component size of 26 mm fits 149 (95.5%) patellae of this series.

Our findings is different from an American study [19] that showed only one-third of their female patients were unable to accommodate their smallest patella implant without overhang. The study also showed that the native patellae which measured about 17 to 21 mm thick may have problem fitting prosthesis between 26 mm and 33 mm in diameter. Thus, in order to avoid over-resection of remaining bone stock and at the same time restore the original patella thickness, the authors suggested prostheses in the diameter range of about 26 to 33 mm need to be available in thicknesses of approximately 5 to 7 mm [19].

Other authors have reported that the patella articular surface is oval, with the width being larger than the height [2–4, 6–8, 10]. The ratio of width to height in this series was 1.31, similar to findings by Aglietti [14], which was also 1.31 and ratio of 1.34 by Zaffagnini et al. [1]. The point that the mean articular surface of patella is 25% wider than its height could be a significant consideration in patella prosthesis design. An American study by Baldwin and House [7] showed that there is potential to improve maximum patellar coverage by 9.1% if the surgeon uses an oval patella implant, with 11.5% improvement of coverage in men and 7.7% in women, which will improve the load transfer through the implant.

Our study relies on an intraoperative direct measurement for the patellae dimension as compared to some studies where measurements were taken from cadavers or from radiographs. Although a radiographic study allows the availability of large sample size, it is usually related to male samples following trauma or sport injury. Furthermore, there is also limitation in accuracy of the measurements due to magnification in radiograph as well as thickness of the patella cartilage that cannot be measured on radiograph. However, for cadaveric study, the limited number of cadavers is the negative point.

There were several limitations present in this study. First, regarding the number of patients involved in this study, we require a larger sample size with multicentre involvement to get better results. Second, a surgical caliper was used to measure the patella dimensions. Even though caliper measurements are commonly used in anthropometric studies, a question raised regarding the accuracy of smooth caliper measurements [20]. Thus, a digital vernier caliper would be a better option. Third, although we consider the effect of pre-arthritic thickness of the patellae involved in this study by eliminating all the deformed and misshapen patellae, the local effect and the extent of patella surface thinning due to arthritis at the time of operation are unknown.

## 5. Conclusion

As a conclusion, the implant manufacturers will have to consider ideal size of patella component for Asian patients, especially for the female. This is to provide optimum patella coverage during the patella resurfacing in a tricompartmental TKA in order to achieve optimum biomechanical integrity of the patellofemoral joint after TKA. The Malaysian female knees have thinner native patella and generally accommodate smaller patellar components than the Caucasian female counterparts.

## Figures and Tables

**Table 1:** Age and dimensions of patellae (*n* = 156).

Variables	Mean (SD)
Age (years)	65.50 (7.41)
Patella thickness (mm)	20.74 (1.85)
Patella height (mm)	31.33 (2.81)
Patella width (mm)	40.76 (3.79)
MAF width (mm)	19.38 (2.70)
LAF width (mm)	21.30 (2.27)
MAF thickness (mm)	15.90 (2.24)
LAF thickness (mm)	16.93 (2.16)
Width-height ratio	1.31 (0.13)

MAF = medial articular facets; LAF = lateral articular facets.

**Table 2:** Comparison of patella dimensions between the right and left side (*n* = 156).

Variables (mm)	Right side, mean (SD)	Left side, mean (SD)	Mean difference (95% CI)	*t* statistics (df)	*p* value
Patella height	31.41 (2.88)	31.24 (2.77)	0.17 (−1.11, 1.45)	0.27 (76)	0.790
Patella width	40.67 (3.62)	40.85 (4.02)	−0.18 (−1.90, 1.54)	−0.21 (76)	0.835
Patella thickness	20.82 (2.06)	20.65 (1.61)	0.17 (−0.67, 1.01)	0.40 (76)	0.691
MF width	19.22 (2.66)	19.55 (2.77)	−0.33 (−1.56, 0.89)	−0.54 (76)	0.588
LF width	21.30 (2.35)	21.30 (2.20)	0.01 (−1.02, 1.04)	0.02 (76)	0.988

MF = medial facet; LF = lateral facet.

**Table 3:** Comparison of patella thickness and width with Wiberg type (*n* = 156).

Variable (mm)	Wiberg type 1, mean (SD) (*n* = 90)	Wiberg type 2, mean (SD) (*n* = 53)	Wiberg type 3, mean (SD) (*n* = 9)	*F*-statistics (d*f*)	*p* value^*∗*^
Patella thickness	20.71 (1.67)	20.81 (2.29)	20.60 (1.14)	0.04 (2)	0.965
Patella width	41.03 (3.81)	40.02 (3.85)	42.00 (3.32)	0.88 (2)	0.418

^*∗*^One-way Anova. Since *p* value is not significant, the post hoc test is not performed.

**Table 4:** Comparison of width-height ratio and patella height between the group of patients suitable for oval-shape implants and round-shape implants (*n* = 156).

Variable	Oval, mean (SD) (*n* = 84)	Round, mean (SD) (*n* = 72)	Mean difference (95% CI)	*t* statistics (d*f*)	*p* value^*∗*^
Width-height ratio	1.40 (0.09)	1.20 (0.06)	0.20 (0.16, 0.23)	11.18 (76)	<0.001
Patella height (mm)	30.18 (2.51)	32.68 (2.56)	−2.50 (−3.64, −1.36)	−4.35 (76)	<0.001

^*∗*^Independent *T*-test.

## Data Availability

The data used to support the findings of this study are included within this article.

## Supplementary Materials

Supplementary Materials Concise description on supplementary files. Figure 1: measurement of left patella's width using a vernier caliper. Figure 2: measurement of left patella's height using a vernier caliper. Table I: descriptive analysis of study: age and dimensions of patellae (*n* = 156). Table II: comparison of patella dimensions between the right and left side (*n* = 156). Table III: comparison of patella thickness and patella width with Wiberg type (*n* = 156). Table IV: comparison of width-height ratio and patella height between the group of patients suitable for oval-shape implants or round-shape implants (*n* = 156).
