# Supplementary material for: Intraoperative Patella Dimension Measurement in Asian Female Patients and Its Relevance in Patellar Resurfacing in TKA
Source: Adv Orthop. 2020 Apr 25;2020:4539792. doi: 10.1155/2020/4539792 (PMC7210516; doi:10.1155/2020/4539792)
Supplement: Supplementary Materials — Concise description on supplementary files. Figure 1: measurement of left patella's width using a vernier caliper. Figure 2: measurement of left patella's height using a vernier caliper. Table I: descriptive analysis of study: age and dimensions of patellae (n = 156). Table II: comparison of patella dimensions between the right and left side (n = 156). Table III: comparison of patella thickness and patella width with Wiberg type (n = 156). Table IV: comparison of width-height ratio and patella height between the group of patients suitable for oval-shape implants or round-shape implants (n = 156). [file 4539792.f1.docx]

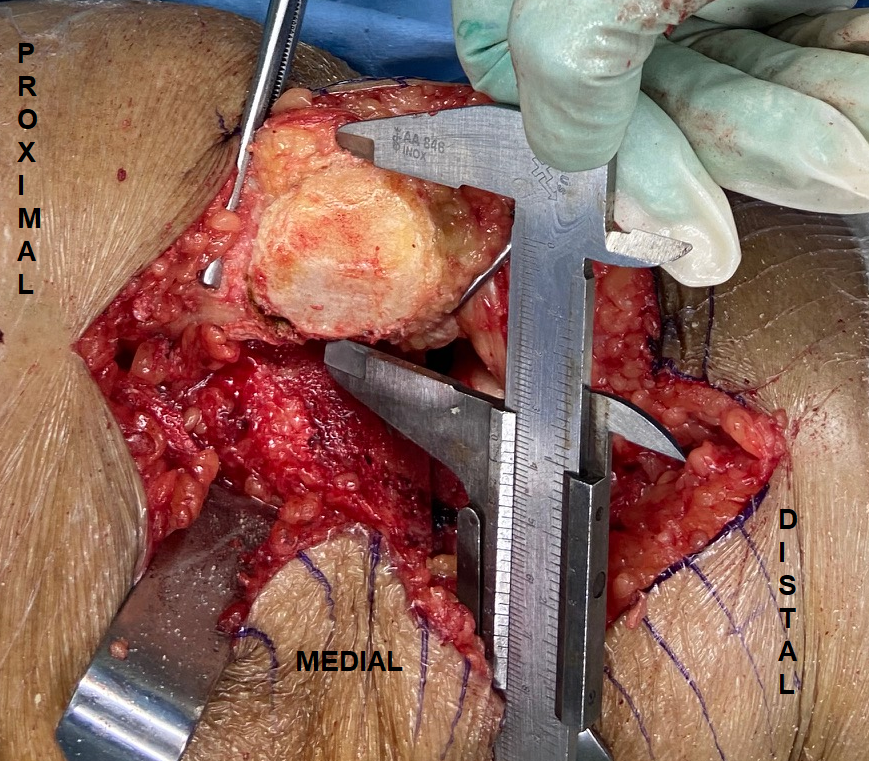


**Figure 1 : Measurement of left patella’s width using a Vernier caliper**


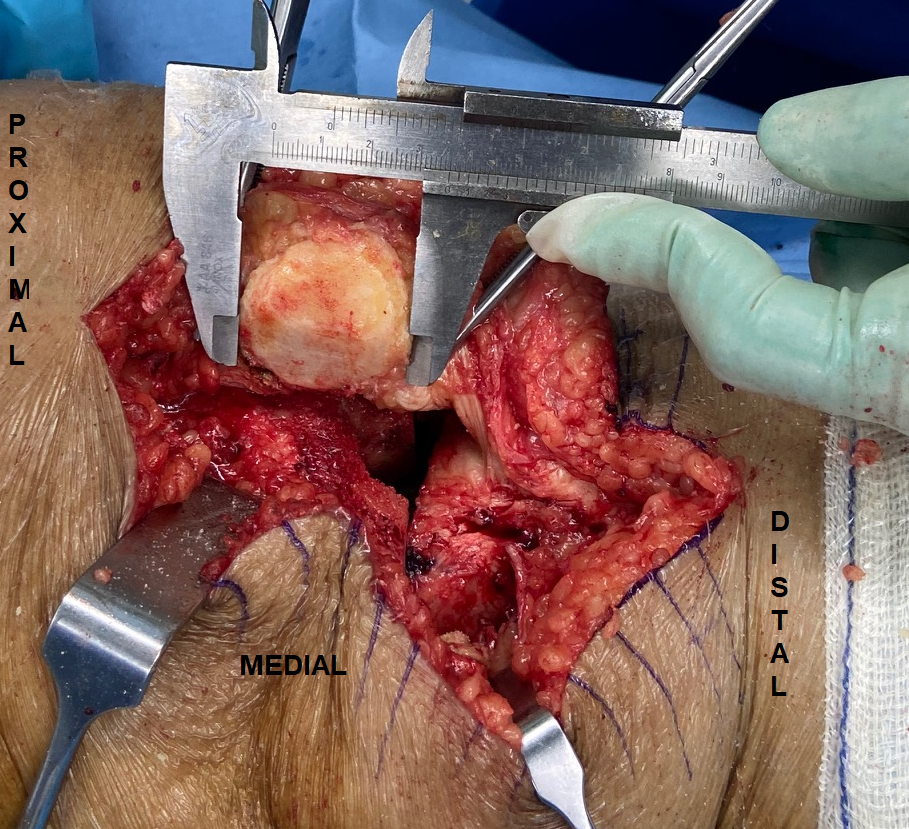


**Figure 2 : Measurement of left patella’s height using a Vernier caliper**

Table I : Age and dimensions of patellae (n=156)

| Variables | Mean (SD) |
| --- | --- |
| Age (years) | 65.50 (7.41) |
| Patella Thickness (mm) | 20.74 (1.85) |
| Patella Height (mm) | 31.33 (2.81) |
| Patella Width (mm) | 40.76 (3.79) |
| MAF Width (mm) | 19.38 (2.70) |
| LAF Width (mm) | 21.30 (2.27) |
| MAF Thickness (mm) | 15.90 (2.24) |
| LAF Thickness (mm) | 16.93 (2.16) |
| Width-height Ratio | 1.31 (0.13) |

MAF = medial articular facets

LAF = lateral articular facets

Table II : Comparison of patella dimensions between right and left side (n=156).

| Variables (mm) | Right side, Mean(SD) | Left side, Mean(SD) | Mean difference (95% CI) | t statistics (df) | p value |
| --- | --- | --- | --- | --- | --- |
| Patella height | 31.41(2.88) | 31.24(2.77) | 0.17 (-1.11, 1.45) | 0.27 (76) | 0.790 |
| Patella width | 40.67(3.62) | 40.85(4.02) | -0.18(-1.90,1.54) | -0.21(76) | 0.835 |
| Patella thickness | 20.82(2.06) | 20.65(1.61) | 0.17(-0.67,1.01) | 0.40(76) | 0.691 |
| MF width | 19.22(2.66) | 19.55(2.77) | -0.33(-1.56,0.89) | -0.54(76) | 0.588 |
| LF width | 21.30(2.35) | 21.30(2.20) | 0.01(-1.02,1.04) | 0.02(76) | 0.988 |

MF = medial facet

LF = lateral facet

Table III : Comparison of Patella thickness and width with Wiberg Type (n=156).

| Variable (mm) | Wiberg Type 1, Mean(SD)  (n=90) | Wiberg Type 2, Mean(SD)  (n=53) | Wiberg Type 3, Mean(SD)  (n=9) | F-statistics (df) | p value^*^ |
| --- | --- | --- | --- | --- | --- |
| Patella thickness | 20.71(1.67) | 20.81(2.29) | 20.60(1.14) | 0.04 (2) | 0.965 |
| Patella width | 41.03(3.81) | 40.02(3.85) | 42.00(3.32) | 0.88 (2) | 0.418 |

^*^One Way Anova

Since p value is not significant, post-hoc test is not performed

Table IV: Comparison of width-height ratio and patella height between group of patients suitable for oval-shape implant and round-shape implant (n=156).

| Variable | Oval, Mean(SD) (n=84) | Round, Mean(SD) (n=72) | Mean difference (95% CI) | t statistics (df) | p value^*^ |
| --- | --- | --- | --- | --- | --- |
| Width-height ratio | 1.40(0.09) | 1.20(0.06) | 0.20 (0.16, 0.23) | 11.18 (76) | <0.001 |
| Patella height (mm) | 30.18(2.51) | 32.68(2.56) | -2.50(-3.64,-1.36) | -4.35(76) | <0.001 |

^*^Independent T-test
